# Uterus *infantilis:* a novel phenotype associated with *AARS2* new genetic variants. A case report

**DOI:** 10.3389/fneur.2023.878446

**Published:** 2023-06-29

**Authors:** Ekaterina Kazakova, José Alberto Téllez-Martínez, Leonardo Flores-Lagunes, Ana Luisa Sosa-Ortiz, Karol Carillo-Sánchez, Carolina Molina-Garay, Carlos Alberto González-Domínguez, Marco Jimenez-Olivares, Francisca Fernandez-Valverde, Edwin Steven Vargas-Cañas, Martha Elisa Vázquez-Memije, Ethel Awilda Garcia-Latorre, Iván Martínez-Duncker, Carmen Alaez-Verson

**Affiliations:** ^1^Centro de Diagnóstico en Metabolismo Energético y Medicina Mitocondrial, Mexico City, Mexico; ^2^Clínica de Cognición, Instituto Nacional de Neurología y Neurocirugía Manuel Velasco Suárez, Mexico City, Mexico; ^3^Laboratorio de Diagnóstico Genómico, Instituto Nacional de Medicina Genómica, Mexico City, Mexico; ^4^Laboratorio de Glicobiología Humana y Diagnóstico Molecular, Centro de Investigación en Dinámica Celular, Instituto de Investigación en Ciencias Básicas y Aplicadas, Universidad Autónoma del Estado de Morelos, Morelos, Mexico; ^5^Laboratorio de Patología Experimental, Instituto Nacional de Neurología y Neurocirugía Manuel Velasco Suárez, Mexico City, Mexico; ^6^Clínica de Nervio y Músculo, Instituto Nacional de Neurología y Neurocirugía Manuel Velasco Suárez, Mexico City, Mexico; ^7^Escuela Nacional de Ciencias Biológicas, Instituto Politécnico Nacional, Mexico City, Mexico

**Keywords:** mitochondrial aminoacyl-tRNA synthetase, *AARS2*, adult-onset leukodystrophy, progressive leukoencephalopathy with ovarian failure, uterus *infantilis*, alanyl-transfer RNA synthetase 2 mutation-related leukodystrophy, early-onset dementia, AARS2 leukoencephalopathy

## Abstract

**Objectives:**

To report the first Mexican case with two novel *AARS2* mutations causing primary ovarian failure, uterus *infantilis*, and early-onset dementia secondary to leukoencephalopathy.

**Methods:**

Detailed clinical, clinimetric, neuroimaging features, muscle biopsy with biochemical assays of the main oxidative phosphorylation complexes activities, and molecular studies were performed on samples from a Mexican female.

**Results:**

We present a 41-year-old female patient with learning difficulties since childhood and primary amenorrhea who developed severe cognitive, motor, and behavioral impairment in early adulthood. Neuroimaging studies revealed frontal leukoencephalopathy with hypometabolism at the fronto-cerebellar cortex and caudate nucleus. Uterus *infantilis* was detected on ultrasound study. Clinical exome sequencing identified two novel variants, NM_020745:c.2864G>A (p.W955^*^) and NM_020745:c.1036C>A (p.P346T, p.P346Wfs^*^18), in *AARS2*. Histopathological and biochemical studies on muscle biopsy revealed mitochondrial disorder with cytochrome C oxidase (COX) deficiency.

**Conclusions:**

Several adult-onset cases of leukoencephalopathy and ovarian failure associated with *AARS2* variants have been reported. To our best knowledge, none of them showed uterus *infantilis*. Here we enlarge the genetic and phenotypic spectrum of *AARS2*-related dementia with leukoencephalopathy and ovarian failure and contribute with detailed clinical, clinometric, neuroimaging, and molecular studies to disease and novel molecular variants characterization.

## Background

The alanyl-tRNA synthetase 2 (*AARS2*, MIM#612035) is a 22 exons nuclear gene located on 6p21.1. It encodes the mitochondria-specific alanyl-tRNA synthetase enzyme, responsible for the aminoacylation between alanine and the tRNA during translation in the mitochondria. The AARS2 protein contains editing and aminoacylation domains. Deleterious variants in these sites affect the catalysis of aminoacylation (1).

Deficiency in aminoacyl-tRNA synthetases is known to contribute to mitochondrial diseases in association with a broad spectrum of clinical phenotypes (2, 3). *AARS2* pathogenic variants were first identified in infantile mitochondrial cardiomyopathy (4) and later found to cause an adult-onset autosomal recessive leukodystrophy, MIM#615889 (https://www.omim.org/) (5).

The classic presentation of alanyl-transfer RNA synthetase-2 mutation-related leukodystrophy (AARS2-L) is a childhood-to-adulthood onset neurologic deterioration primarily related to frontal and cerebellar dysfunction that manifests with ataxia, spasticity, cognitive decline, psychiatric disorders or executive dysfunction (6). MRI signal abnormalities are primarily found in frontal and parietal white matter and the corpus callosum. There is no established treatment for AARS2-L (5).

To our best knowledge, 27 index AARS2-L cases have been reported up to date (5, 7–14). Amazingly, most described cases are adult-onset with nearly invariable progression to severe disability and atrophy of the involved brain regions, often within a decade (5–7, 10).

## Case presentation

Here we present the case of a 41-year-old female born to non-consanguineous Mexican parents. The patient has a family history of late-onset Alzheimer's disease (maternal grandmother), alcohol and drug abuse (father and elder brother), major depressive disorder, and a suicide attempt (mother). There is no further information on the father's family history.

The patient reports sexual abuse on one occasion at 13 years, with no medical or psychiatric attention. She has had a history of migraines since adolescence, treated with unspecified non-steroid anti-inflammatory drugs. The patient presented primary amenorrhea; therefore, a pelvic ultrasound was performed at 15 years, reporting uterus *infantilis* with atrophic endometrium. Menarche was hormonally induced at 15 years. The patient has never become pregnant.

From the age of 35 years, she is reported to have multiple sexual partners, and at 40, she began with a cognitive and behavioral impairment that resulted in a notable work-related dysfunction. The relative found her under deplorable hygienic conditions and crude self-care. However, she preserved partial functionality that allowed the completion of basic activities like bathing and feeding under supervision while losing the ability to carry out instrumented activities of daily life. At 41 years old, she was diagnosed with type I bipolar disorder and treated with quetiapine 100 mg/day and fluoxetine 20 mg/day, but no improvement was observed.

Since the deterioration progressed, at 41 years, she attended INNN and was evaluated by the Dementia Clinic. The most relevant clinical findings include: a puerile attitude, disorientation in space and time, emotional liability and pathological crying, inattention, altered speech (phonological paraphasias, substitutions, and perseverations), difficulty in understanding complex orders, perseverative behavior, increased psychomotricity due to anxiety, environmental dependence, and anosognosia.

**Figure 1:**
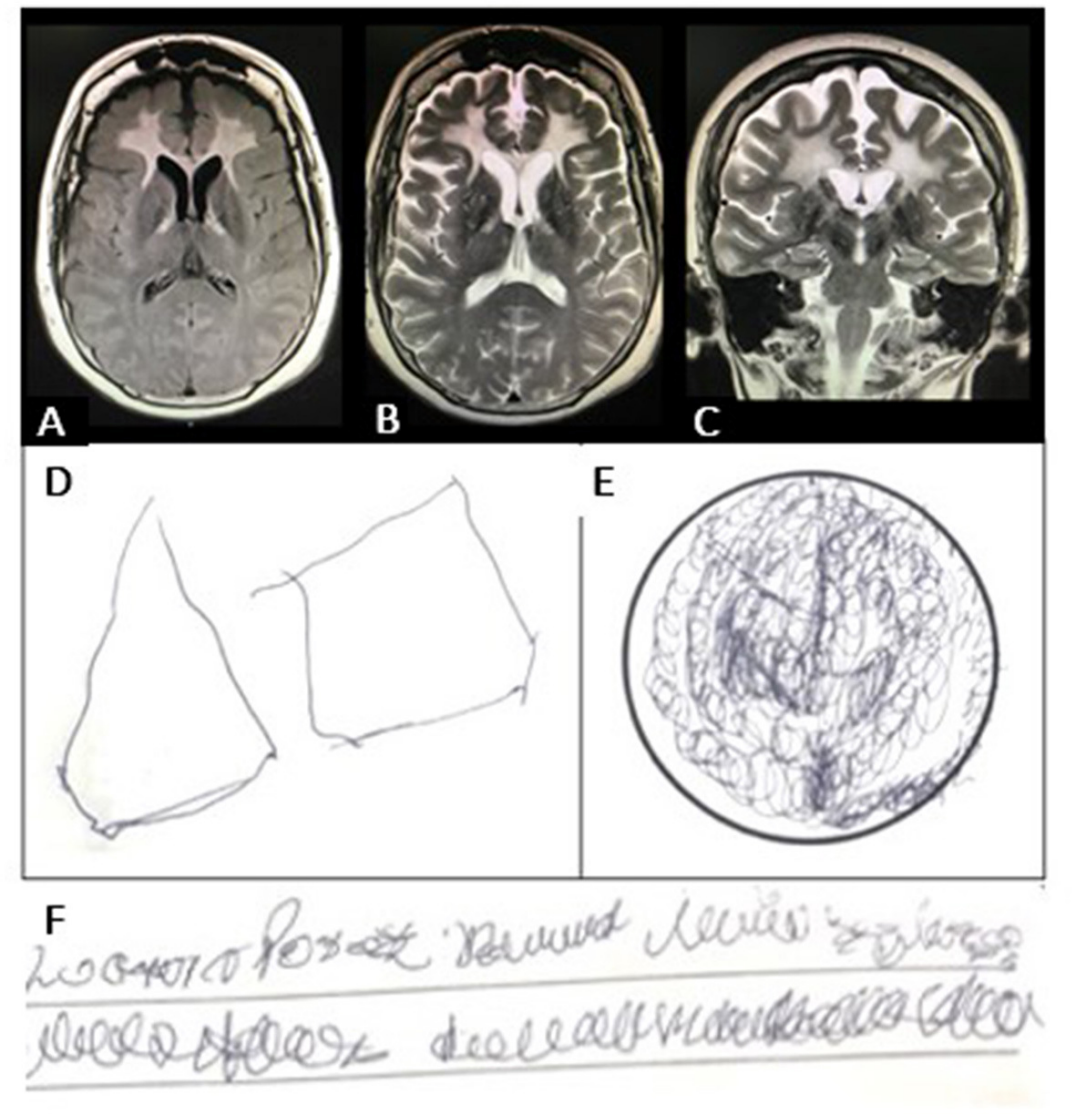
**(A)** Brain magnetic resonance imaging (MRI) shows abnormal signals in the bilateral periventricular anterior white matter and the anterior part of the corpus callosum. **(B)** Axial T2-weighted MRI shows abnormal signals in the frontal white matter and in the pyramidal tract at the internal capsule. There was no clear pattern of cortical atrophy, and the hippocampal volume was normal. **(C)** The diffusion-weighted image (DWI) shows patchy areas of restricted diffusion in the abnormal white matter. **(D)** Visuospatial ability performance in the Minimental Status Examination (MMSE) test showing visuospatial disintegration (no intersection, loss of some sides and angles); **(E)** Clock Drawing Test (CDT) with perseverative drawing; **(F)** Writing a sentence in the MMSE test showing dysgraphia.

On physical examination, cardiovascular alterations were absent, and pyramidal signs and frontoparietal dysfunction were present. Laboratory tests excluded infectious, toxic-metabolic, neoplasm, and endocrinologic etiologies. No alterations were detected in cerebrospinal fluid analysis, electroencephalographic studies, and karyotype. The brain magnetic resonance imaging (MRI) showed abnormal signals in the bilateral periventricular anterior white matter and the anterior part of the corpus callosum (Figures 1A–C). 18-F FDG PET/MRI showed frontal leukoencephalopathy with hypometabolism at the fronto-cerebellar cortex and caudate nucleus. The performance in cognitive tests and neuropsychiatric evaluation reports are shown in Supplementary Table 1 and Figures 1D–F.

**Figure 2:**
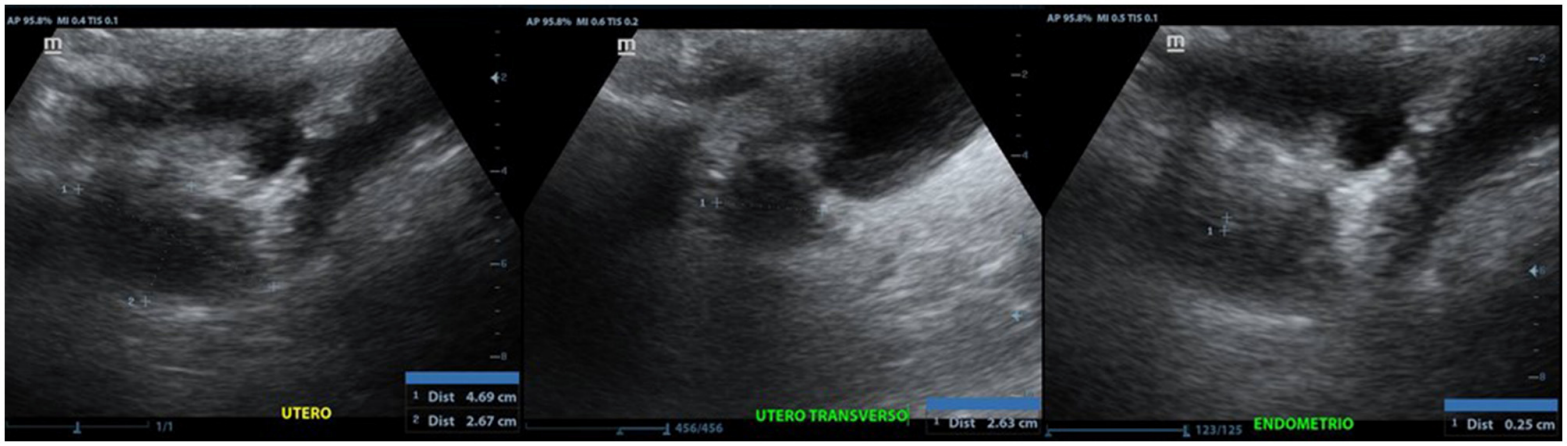
Anteverted uterus, with regular and poorly defined contours, with approximate dimensions of 4.6 x 2.6 x 2.6 cm in its longitudinal, transverse and anteroposterior axes, respectively.

At 46 years, due to the previous uterus *infantilis* report, an additional sonographic study on the pelvic region was performed. A Mindray Z6 computerized system and a multi-frequency convex transducer were used. The study revealed a diminished size uterus confirming the uterus *infantilis* (Figure 2).

Supportive treatment was initiated with citalopram 30 mg/24 h, quetiapine 200 mg/24 h, and gait rehabilitation. Some neuropsychiatric symptoms, mainly emotional lability, psychotic symptoms, and behavioral disturbances, were gradually improved. However, the patient continued rapidly deteriorating cognitive and motor aspects, and a complete loss of autonomy occurred at 43.

### Genetic analysis

#### Clinical exome sequencing

DNA was extracted from peripheral blood. According to the manufacturer's protocol, library preparation was performed using the reagents provided in the Clinical Exome Sequencing panel kit, version 2 (Sophia Genetics SA, Saint Sulpice, Switzerland). The panel includes coding regions and intron/exon borders of 4,900 genes. Sequencing was performed on NextSeq Instrument (Illumina San Diego, CA). Sequencing data analysis and variant annotation were performed with the Sophia DDM^®^ platform (Sophia Genetics SA, Saint Sulpice, Switzerland). Copy number variations were predicted from the sequencing data. Virtual panels were built, including the known genes associated with major neurocognitive disorders. Information from variant databases and international literature was used in the variant review process. Two novel variants were identified in the *AARS2* gene: NM_020745:c.1036C>A (p.P346T) and NM_020745:c.2864G>A (p.W955^*^).

#### Sanger sequencing confirmation

**Figure 3:**
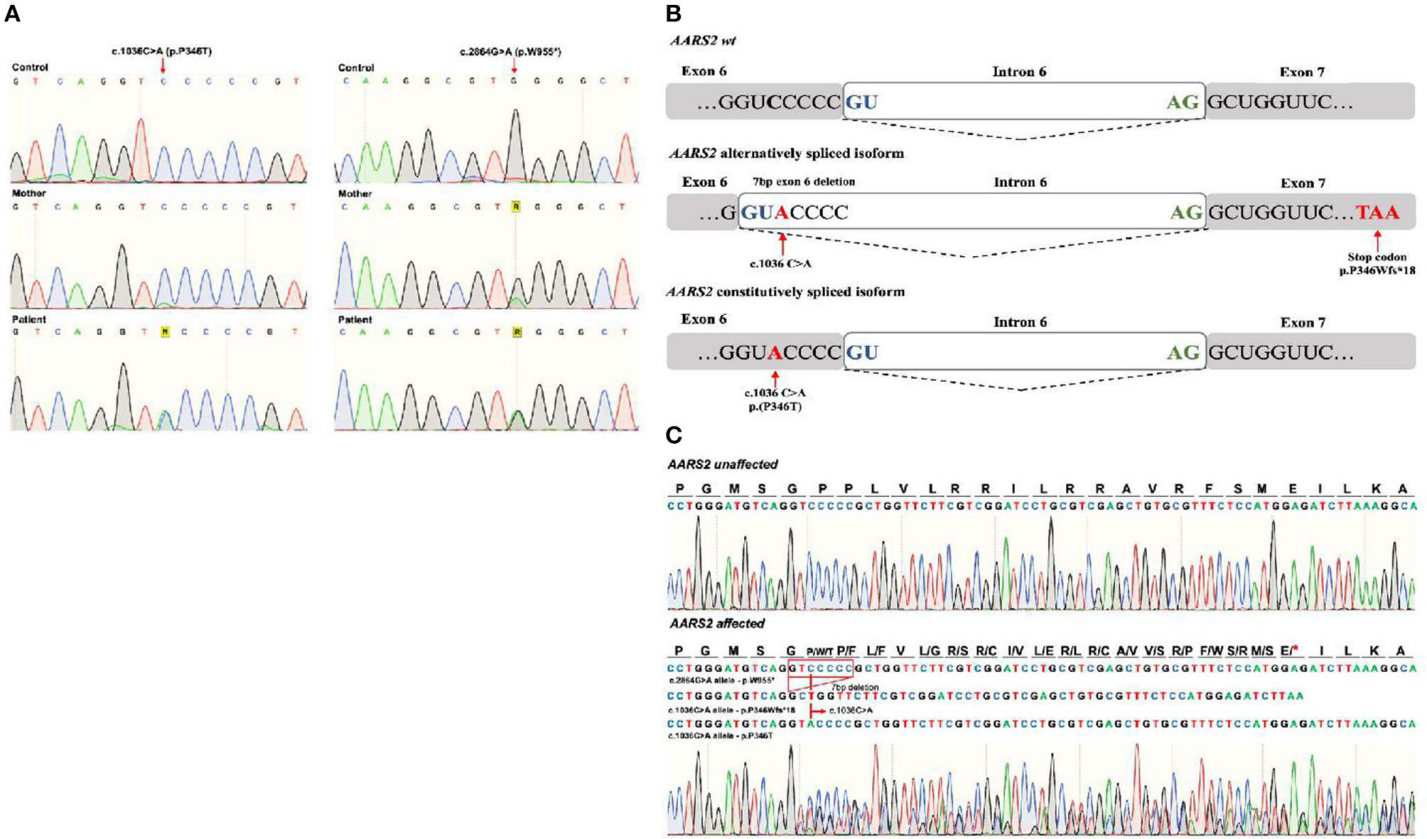
**(A)** Sanger sequencing of the *AARS2* in gDNA from the control, mother, and patient confirming the presence of both variants: NM_020745:c.1036C>A (p.P346T) and NM_020745:c.2864G>A (p.W955^*^) in the patient. The p. c.2864G>A ^*^ was identified in the mother. **(B)** Schematic representation of a wild type, and the alternative and constitutive splicing isoforms caused by the c.1036C>A variant. **(C)** Sanger sequencing showing the pathogenic alternative splicing caused by the c.1036C>A variant. Three PCR products were sequenced from a unique PCR band amplifying the *AARS2* transcript. The corresponding sequences are shown in the AARS2 affected” section of the figure: the first corresponds to the allele with the c.2864G>A, p.W955^*^ variant that appears normal in the shown area. The second read showed a deletion of seven exon nucleotides that remove the c.1036C>A and causes a change in the reading frame with a premature stop codon as a predicted consequence (p.P346Wfs^*^18). The third read corresponds to the c.1036C>A constitutively spliced variant translating as a P346T aminoacid substitution without a change in the reading frame.

In order to confirm the identified variants, gDNA-polymerase chain reaction (PCR) products from the corresponding segments of the *AARS2* exons 6 and 22 were obtained. For exon 6 primers: F-AARS2-E6s 5′-CTGGATGTGGTTGGCTTTAGGG-3′ and R-AARS2-E6as 5′-CCAGTGTGTGCTCCCACCTC-3′ were used. For exon 22 F-AARS2-E22s 5′-CACTCTTGAGGGTACCTTG-3′ and R-AARS2-E22as 5′-CAGCCTCTAGTCCTCGC-3′ primers were used. Direct Sanger sequencing of PCR products was carried out. Electrophoretic analysis was performed on ABI Prism 3130xl genetic analyzer (Applied Biosystems, Foster City, CA). Both variants were confirmed by Sanger sequencing in the patient. The variant c.2864G>A (p.W955^*^) was also identified in the patient's healthy mother (Figure 3A). No DNA from the father was available to complete the segregation analysis.

#### Variant classification

NM_020745:c.2864G>A is a nonsense variant in exon 22 that originates a premature stop codon p.W955^*^. This change is predicted to cause protein loss of 31 amino acids. The variant is absent from dbSNP, 1,000 Genomes Browser phase 3, ExAC, and gnomAD (exomes and genomes) databases. It is not listed in the ClinVar database nor the professional Human Gene Mutation Database (HGMD) (last accessed Nov 2022 for all databases). We classified p.W955^*^ as pathogenic according to the American College of Medical Genetics (ACMG) criteria.

The variant NM_020745:c.1036C>A is a missense variant located in exon 6, that originates p.P346T amino acid substitution. Its population frequency is very low (frequency = 0.0000289) and has been identified in only one Latino individual, according to the gnomAD exomes database. It is absent from gnomAD genomes, 1,000 genomes, ExAC databases, and from a publicly available database that includes 480 exomes from Mexican Mestizo individuals (https://franklin.genoox.com/clinical-db/home). The variant is identified in the dbSNPs with rs1438347145. It does not have a clinical classification assigned in the ClinVar database and is not listed in the HGMD database (last accessed Nov 2022).

**Figure 4:**
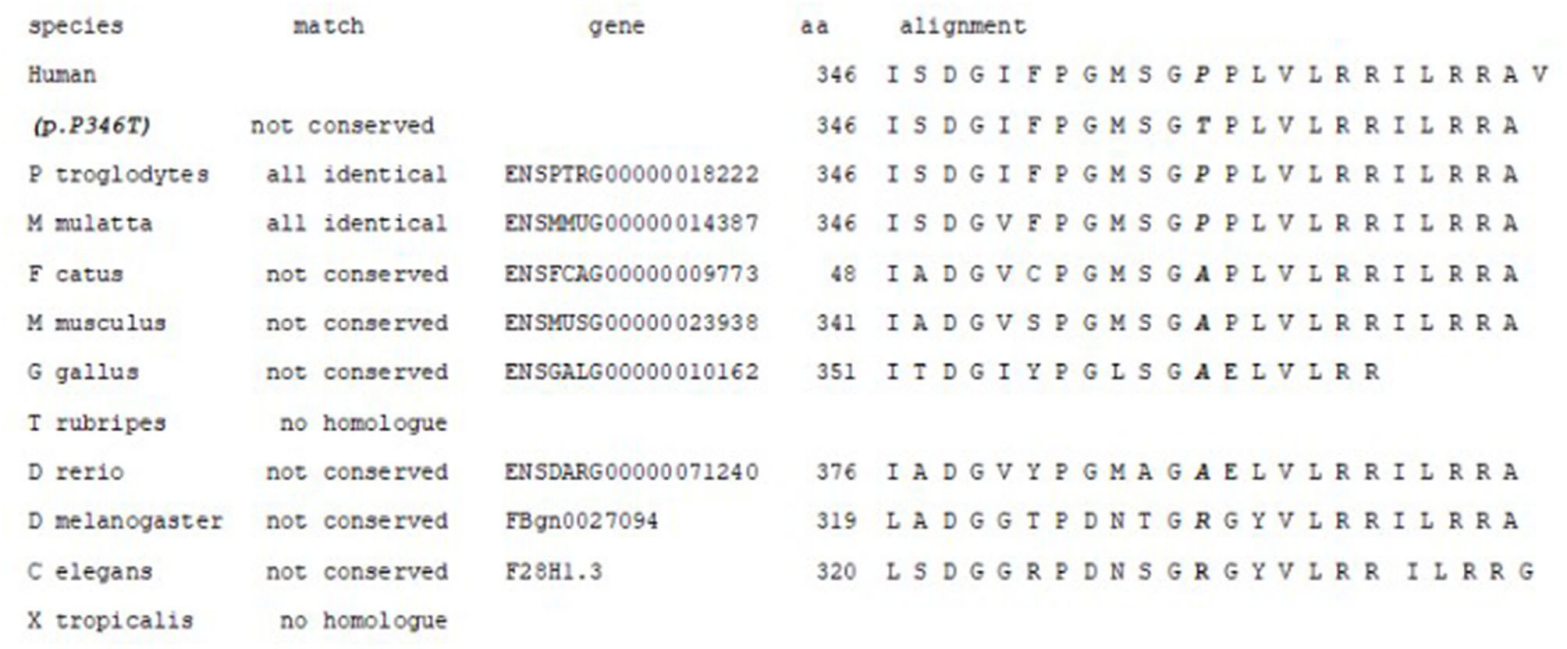
Amino acid alignment of the *AARS2* homologous proteins. The amino acid p.P346 is not conserved; only *Ptroglodytes* and *M. Mulatta* have proline in this position.

Amino acid alignment of the *AARS2* homologous gene is shown in Figure 4. The amino acid p.P346 is not highly conserved; only *Ptroglodytes* and *M. Mulatta* have proline in this position. The change from proline to threonine is not conservative (cyclic-no polar to aliphatic-polar). Predictions from *in silico* algorithm are conflicting: DANN, FATHMM-MKL, LIST-S2, M-CAP, MutationTaster, and SIFT predict as pathogenic while BayesDel_addAF, DEOGEN2, EIGEN, MVP, MutationAssessor and PrimateAI predict as benign.

However, the affected nucleotide (c.1036C) is conserved (PhiloP 2.745 and PhastCons 0.991). It is located five bases away from the canonical splicing site in the exon-intron border. The Mutation Taster algorithm predicts that this change would affect mRNA processing creating a new splicing donor site at position gDNA 6070 (score 0.86) that would affect the zinc-binding domain of the protein. The Human Splicing Finder program also predicts a possible alteration of the splicing (15).

#### Evaluation of possible alteration in splicing

Since variant c.1036C>A was predicted to affect mRNA processing, Sanger sequencing of cDNA-based PCR product of the coding sequence of *AARS2* was performed. The mRNA was obtained from the patient peripheral blood sample using RNeasy Mini Kit (QIAGEN N.V., Hilden, Germany). The primers used were: F-AARS2s 5′-ACACTGACCTCTTTTCCCCG-3′ and R-AARS2as5′-GCCCATGTCTCCTTGTGTCA-3′. The schematic representation and the results are shown in Figures 3B, C. Sanger sequencing revealed that the c.1036C>A variant causes both constitutive splicing harboring the p.P346T change and an alternative splicing caused by the activation of a cryptic donor splice site leading to deletion of seven nucleotides from exon 6, causing a frameshift and a premature stop codon (P346Wfs^*^18). These results confirm that the c.1036C>A originates leaky splicing.

#### Variant analysis for uterus *infantilis*

In order to establish if this feature would be related to pathogenic variants in known causative genes, virtual panels were built using the following Human Phenotype Ontology (HPO) terms: HP:0008684:Aplasia/hypoplasia of the uterus, HP:0000130:Abnormality of the uterus, HP0000013:Hypoplasia of the uterus and, HP0031105:Abnormal uterus morphology. No pathogenic o likely pathogenic variants in the sequenced genes were identified. The analyzed genes are listed in Supplementary material S1.

### Muscle biopsy and mitochondrial respiratory chain findings

**Figure 5:**
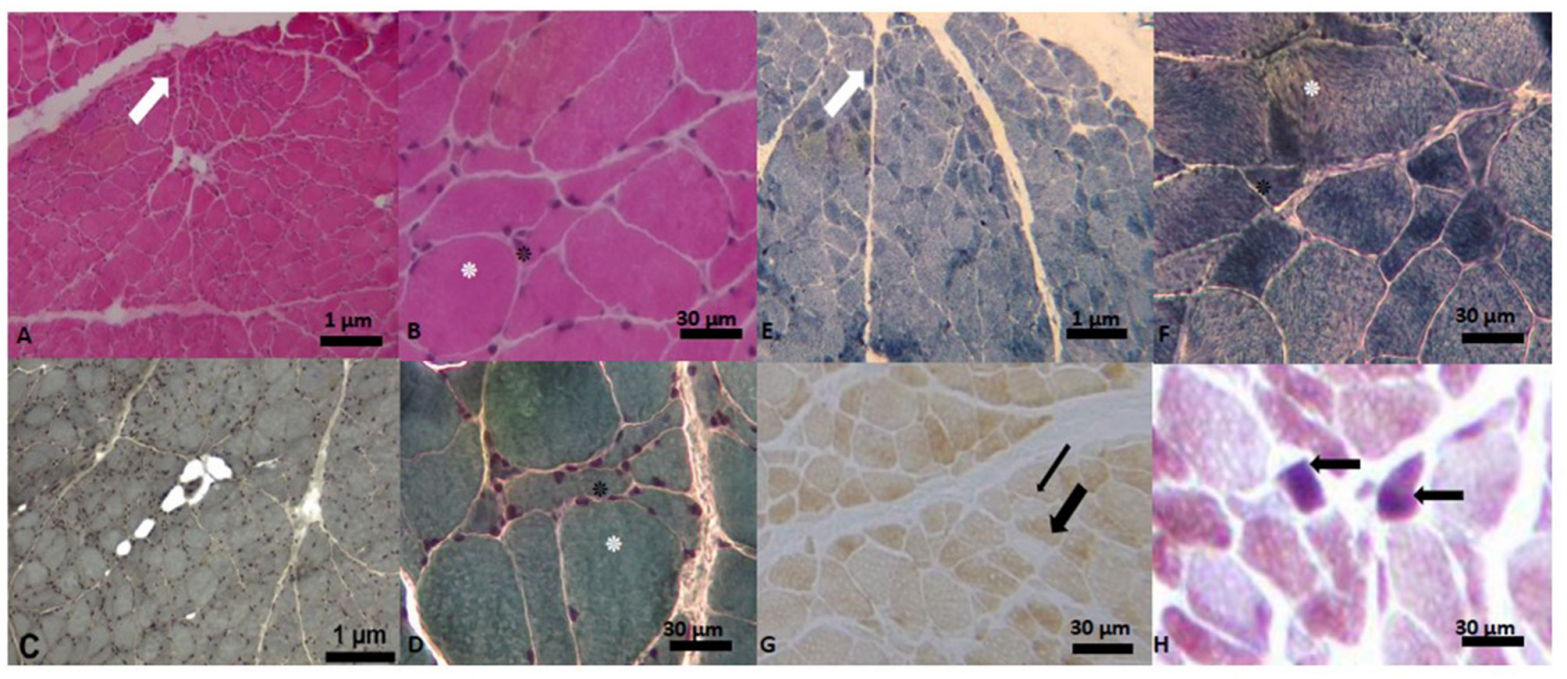
Muscle biopsy images. **(A)** Hematoxylin-Eosin Stain (H-E). Panoramic cross-sectional view of muscle with myopathic and angulated fascicles (marked with white arrow), difference in shape and size of the fiber, intra- and inter-fascicular connective tissue. In **(B)**, close-up of image **(A)** with the presence of the hypertrophic fiber (white asterisk), surrounded by angulated fibers (black asterisk). In **(C, D)** images (Gomori's Modified Trichrome Stain (T-G)) the same findings are observed as in **(A)**. In **(E)** panoramic view with the technique of Reduced Nicotinamide Adenine Dinucleotide-Tetrazolium Reductase (NADH-TR) with type I fibers of darker color and type II fibers of lighter color. In **(F)** close-up of the previous image, where a hypertrophic fiber is observed, surrounded by some angulated fibers. In **(G)**, view of a transverse section with cytochrome C oxidase (COX) showing pale (thin black arrow) and ghost (thick black arrow) fibers. In **(H)** Cytochrome C Oxidase/Succinate Dehydrogenase (COX/SDH) double-labeling method we observe two blue fibers.

At 46 years of age, the patient underwent a left deltoid muscle biopsy. Muscle morphological studies revealed myopathic and neuronopathic changes with loss of COX activity in some fibers and blue fibers presence in Cytochrome C Oxidase/Succinate Dehydrogenase (COX/SDH) double-labeling method which suggests COX deficiency (Figure 5).

Biochemical evaluation of the mitochondrial respiratory chain function was performed by measuring citrate synthase, cytochrome c oxidase, and other mitochondrial enzyme activities in skeletal muscle homogenates, as previously described (16). A complex IV (COX) deficiency was confirmed. Decreased citrate synthase activity was detected, indicating a significant reduction of the mitochondrial content. The activity of other mitochondrial complexes (expressed as nmol/min per mg protein) was also reduced, but after correcting for citrate synthase activity, they were within the normal ranges (Supplementary Table 2).

## Discussion

We report the first Mexican case of AARS2-L, a hereditary autosomal recessive metabolic dementia form with progressive leukoencephalopathy and ovarian failure. AARS2-L is an ultrarare disease with <40 cases described worldwide. The clinical onset usually occurs in the 3rd or 4th decade of life with signs and symptoms originating from fronto-cerebellar dysfunction, including cognitive, behavioral, and motor involvement (5, 17). Spasticity is the most consistent finding, and dystonia, dysarthria, or tremor may also be present (7, 10, 18). All adult-onset patients reported thus far, except two (13, 19), presented leukoencephalopathy. Typically, these patients advance to the point of no or limited interaction with the environment, non-ambulatory status, and in many cases, premature death within 5–10 years after the onset of the symptoms (7, 10, 18). The patient reported here had learning difficulties from childhood, and behavioral and cognitive disturbances began at a young age (apparently at 40 years), causing detriment of self-care and social and work impairment.

All female patients described so far presented primary or secondary ovarian failure except one (20). The ovarian failure in our patient might be the cause of the primary amenorrhea. To our best knowledge, neither reproductive organs nor uterus *infantilis* malformations have been previously described in AARS-L. In our patient, uterus *infantilis* was confirmed by two independent pelvic ultrasound imaging studies.

Uterine hypoplasia is a major malformation due to incomplete morphogenesis. It has been reported in patients with syndromic ciliopathies (MIM#616258, #236700, #236680, #209900), Woodhouse–Sakati syndrome (MIM#241080), basal cell nevus syndrome (Gorlin syndrome) (MIM#109400), Turner syndrome and in Al-Awadi Raas-Rothschild syndrome (21).

Several genes have been related to the proper functioning of the female reproductive system, but challenges exist to prove causality for specific phenotypes (22). Alterations in *DHH, MCM8, MCM9, AMHR2, AMH, LMNA, DCAF17, NR5A1, STAG3*, and *PTPRF* genes have been associated with this malformation. However, in our patient, no pathogenic variants were identified in these genes, nor in other sequenced genes related to the phenotypes:HP0000130: Abnormality of the uterus; HP0031105:Abnormal uterus morphology; HP0008684: Aplasia/hypoplasia of the uterus, HP0000013: Hypoplasia of the uterus.

The *AARS2* gene is highly expressed in the uterus (mean Transcripts Per Million, TPM = 17.97), being overcome only by expression in the ovary (TPM = 20.42), cerebellum (TPM = 20.47) and cerebellar hemisphere (TPM = 21.97) (https://gtexportal.org/home/gene/AARS2). Therefore we might hypothesize that the uterus hypoplasia in this patient would be caused by *AARS2* pleiotropic effect. However, we can not completely rule out a causal pathogenic variant in genes not included in the clinical exome panel (Supplementary material S1).

A multidisciplinary diagnostic approach was performed, excluding the most common neurodegenerative diseases, like Alzheimer's, frontotemporal dementia, autoimmune encephalopathies, prion disease, infections, neoplasms, toxic-metabolic and vascular entities. The integration of clinical, laboratory, and neuroimaging findings, specifically the glycolytic uptake pattern on the F18-FDG PET and the white matter hyperintensities on the MRI, oriented to the diagnosis of leukoencephalopathy.

Massive parallel exome sequencing is a powerful diagnostic tool to identify the affected gene in diseases with high genetic and allelic heterogeneity, like leukoencephalopathies. This approach allows us to establish the etiologic diagnostic in our patient. Two novel deleterious variants in the *AARS2* gene are causing the disease: (NM_020745:c.2864G>A, p.W955^*^) and (NM_020745:c.1036C>A, P346T/P346Wfs^*^18) both of them confirmed by Sanger sequencing. According to HGMD professional database (last accessed Nov 2022), only 69 disease-causing variants have been identified so far; our results extended the mutational spectrum for this gene.

Loss of function of *AARS2* is a known disease mechanism; at least 25 pathogenic null variants have been reported across different exons, including the last one (exon 22) (https://my.qiagendigitalinsights.com/bbp/view/hgmd/pro/all.php). The variant NM_020745:c.2864G>A is a nonsense variant that originates a premature stop codon p.W955^*^, which causes the loss of 31 amino acids in the protein. Kamps et al. reported a patient with severe cardiomyopathy, early onset brain disease and oxidative phosphorylation deficiency due to a compound heterozygous pathogenic variant in AARS2: p.R592W and p.Arg958^*^ (HGMD ID: CM1822327) (23). The last variant is located in exon 22 and produces the loss of 28 aminoacids at the end of the protein, similar to p.W955^*^ identified in the patient reported here, causing a 31 aminoacid loss.

The variant NM_020745:c.1036C>A was predicted to alter the normal splicing of the AARS2 transcript by *in silico* algorithms. cDNA-PCR Sanger sequencing analysis confirmed these predictions and demonstrated that the variant activated a cryptic splice site in exon 6 and produced leaky splicing. Two alternative isoforms were identified: one causing a loss-of-function protein due to seven nucleotide deletions that alter the reading frame originating a premature stop codon (p. P346Wfs^*^18), and the other coding a p.P346T amino acid substitution.

Loss-of-function variants in both alleles of AARS2 may be lethal due to the severe impairment in the mitochondrial protein translation process and mitochondrial functioning. Therefore we speculated that the p.P346T protein might retain some enzymatic activity. In support of that idea, none of the previously identified patients were homozygous or compounded heterozygous for loss-of-function variants, supporting that some residual AARS2 activity is needed to be compatible with life. Additionally, the constrain metrics for the *AARS2* gene in gnomAD database show a significant reduction in the number of loss of function variants observed in the population (55.3 expected vs. 35 observed; o/e = 0.63 (0.48–0.83) (https://gnomad.broadinstitute.org/gene/).

The clinical features presented by our patient support the diagnosis of AARS2-L with mitochondrial myopathic alterations. The mitochondrial abnormalities in histological studies may be very mild and even absent in genetically confirmed mitochondrial diseases, including AARS2-L (24). Muscle biopsies were not performed in all AARS2-L reported cases, but when available, the findings have included normal reports, ragged red fibers, and isolated COX deficiency (9, 24). In the patient we describe, the skeletal muscle biopsy showed COX-negative fibers suggesting a complex IV deficiency confirmed by the biochemical studies. COX is a multimeric enzyme composed of subunits encoded by both nuclear and mitochondrial genomes. Thus, pathogenic variants on the *AARS2* gene play a crucial role in mitochondrial translation and may affect mitochondrial protein synthesis and the functioning of the respiratory chain complexes (24). Ragged red fibers, generally associated with mitochondrial proliferation, were not detected. Still, in contrast, decreased citrate synthase activity, which indicates a reduction of the mitochondrial content, was found, probably related to the late clinical stage of this patient.

There are no specific treatments or disease modifiers for AARS2-L. The use of symptomatic therapy (antidepressants, antipsychotics), as in the case of the patient we present, can help to control some of the neuropsychiatric manifestations and relieve the burden of family/caregivers.

## Conclusions

Inherited metabolic disorders are a rare etiology of early-onset dementias but should be considered in the diagnostic approach. The correlation with clinical findings and molecular biomarkers, including structural and functional neuroimaging and genetic analysis, allows us to establish the aetiologic diagnosis, which is particularly important in high heterogeneity phenotypic and genotypic diseases. In this work, we describe the first Mexican patient with the AARS2-L and characterize two novel variants of the *AARS2* gene. We might hypothesize that the uterus *infantilis* in this patient would be caused by the *AARS2* deficiency pleiotropic effect, but we cannot exclude other causative genes or etiology. This report contributes to enlarging the phenotypic and genetic spectrum of *AARS2* variants and widening the knowledge of early-onset dementias associated with leukoencephalopathy.

## Author's note

In “Uterus Infantilis: a novel Phenotype Associated with AARS2 New Genetic Variants. A Case Report,” we describe a Mexivcan patient showing two novel pathogenic variants in the *AARS2* gene and we enlarge the phenotype. *AARS2* encodes mitochondrial alanyl-tRNA synthetase, which is responsible for the aminoacylation between alanine and the tRNA during translation in the mitochondria. *AARS2* pathogenic variants were first identified in infantile mitochondrial cardiomyopathy in 2011 (4), and later found to cause adult-onset leukodystrophy, MIM#615889 (5). All adult-onset patients, less than two, present leukoencephalopathy (13, 19). All female patients, less than one (20) present ovarian failure, none has been described with uterus *infantilis*. With our paper, we inform the clinician about the detailed psychiatric involvement and describe the reproductive organs alteration possibly associated with *AARS2* related leukodystrophy. We describe two novel compound heterozygous pathogenic variants presenting with learning difficulties from childhood and primary amenorrhea, severe rapidly progressive cognitive, motor and behavioral impairment developed in early adulthood and uterus *infantilis*. Neuroimaging studies revealed frontal leukoencephalopathy with hypometabolism at the fronto-cerebellar cortex and caudate nucleus. Muscle biopsy and enzymatic activity of the respiratory chain, determined in a skeletal muscle biopsy sample, showed mitochondrial abnormalities pointing to the COX. We might speculate that the uterus hypoplasia presented by this patient is caused by a *AARS2* pleiotropic effect, but we cannot definitely exclude that other causative gene is involved. We think that the description of our case might help diagnose unsolved cases of early-onset dementia with leukodystrophy. We alert clinicians to consider inherited metabolic diseases as a possible cause of dementia.

## Data availability statement

The datasets presented in this article are not readily available because of ethical and privacy restrictions. Requests to access the datasets should be directed to the corresponding author.

## Ethics statement

The studies involving human participants were reviewed and approved by Instituto Nacional de Neurología Manuel Velasco Suarez Ethical Review Board for clinical studies. The patients/participants provided their written informed consent to participate in this study. Written informed consent was obtained from the participant/patient(s) for the publication of this case report.

## Author contributions

EK conceived the study and design, analyzed the family history, (clinical, genetic, histopathological and biochemical) data of the patient, conceived the manuscript, wrote and drafted the manuscript, coordinated the collection and elaboration of blood samples data (genetic, histopathological, and biochemical), and contributed to the performing of the analysis of the molecular data. JT-M made the clinical diagnosis, recruited the patient, analyzed the clinical, neuropsychological, and imaging data of the patient, provided the images for Figure 1 and Table 1, conceived the manuscript, contributed to the writing of the case presentation and case presentation part of the discussion of the manuscript, read, and approved the manuscript. LF-L performed the clinical exome genetic analyses, contributed to genetic analysis (clinical exome sequencing, variant classification) part of the manuscript, read, and approved the manuscript. AS-O analyzed the clinical, neuropsychological, and imaging data of the patient, conceived the manuscript, provided patient's clinical data, coordinated the collection and elaboration of patient's data, supervised the study, contributed to the writing of the background, case presentation part of the discussion and conclusions the manuscript, drafted the manuscript, supervised the hole study project, read, and approved the manuscript. KC-S performed the clinical exome genetic analyses and contributed to genetic analysis (clinical exome sequencing, variant classification) part of the manuscript, read, and approved the manuscript. CM-G performed the clinical exome genetic analyses, contributed to genetic analysis (clinical exome sequencing, variant classification) part of the manuscript, contributed to genetic analysis part of the manuscript, read, and approved the manuscript. CG-D performed the segregation and mRNA studies, contributed to the evaluation of possible alteration in splicing part of the manuscript and related part of the discussion, read, and approved the manuscript. MJ-O performed the clinical exome genetic analyses and contributed to genetic analysis (clinical exome sequencing, variant classification) part of the manuscript. FF-V performed the histopathological studies, provided the histopathological images for Figure 5, contributed to histopathological studies interpretation, wrote the Muscle biopsy findings part of the manuscript, as well as to the related discussion of this findings in the manuscript, read, and approved the manuscript. EV-C performed the muscle biopsy and histopathological studies interpretation, revised the muscle biopsy findings part of the manuscript as well as the related discussion of this findings in the manuscript, read, and approved the manuscript. MV-M performed the enzymatic activity of the mitochondrial complexes of the respiratory chain, provided Table 2, wrote the mitochondrial respiratory chain findings part of the manuscript as well as the related discussion of this findings in the manuscript, read, and approved the manuscript. EG-L supervised the study, contributed to the writing of the background, discussion and conclusions parts of the manuscript, supervised the Ph.D. degree work, read, and approved the manuscript. IM-D performed the segregation and mRNA studies, provided the images for Figure 3, and wrote the evaluation of possible alteration in splicing part of the manuscript and related part of the discussion, read, and approved the manuscript. CA-V conceived the study and design, coordinated and supervised the performing of molecular studies (clinical exome sequencing), coordinated the performing of the Sanger sequencing and RNA studies, performed the analysis of the molecular genetic data (variant classification and analysis, variant analysis for uterus infantilis), contributed to the evaluation of possible alteration in splicing, wrote the manuscript, contributed to the editing the English version, contributed to the writing of the background, genetic analysis, discussion, and conclusions of the manuscript.
