# Supplementary material for: Uterus infantilis: a novel phenotype associated with AARS2 new genetic variants. A case report
Source: Front Neurol. 2023 Jun 29;14:878446. doi: 10.3389/fneur.2023.878446 (PMC10343430; doi:10.3389/fneur.2023.878446)
Supplement: Supplementary file 2 [file Data_Sheet_1.PDF]

Table 1. Performance in cognitive tests and neuropsychiatric evaluation report		
Clinimetric evaluation	MMSE	15/30
	Semantic fluency (animals):	3 + 1 (perseveration)
	Phonological fluency (p letter):	1 + 2 (perseveration)
	CDT	0/4
	Functional Activities Questionnaire	24/30
	Sleep Disorders Inventory	Daytime sleepiness, snoring
	Neuropsychiatric Inventory	Anxiety, apathy/indifference, irritability, motor dysfunction, insomnia, hyperphagia
	IQ-Code	4.88
Neuropsychological evaluation	Orientation	Temporal and spatial disorientation
	Attention	Difficulty focusing and maintaining attention
	Memory	Moderate alteration in registration and evocation
	Language	Decrease in verbal fluency, speech with numerous phonological paraphasia's, as well as substitutions and perseverations, difficulty understanding complex orders, alteration in the nomination of objects of infrequent use and low familiarity, alteration in the repetition of long sentences and of greater grammatical complexity
	Abstraction	Alteration in the capacity for abstraction and concrete thinking
	Executive functions	Difficulty integrating, planning, facilitation and adaptation to new situations, as well as for formulation of new action plans, selection and programming of appropriate response sequences for any task
	Visoconstructive skills	Mild impairment in visoconstructive skills
Conclusion	Moderate to severe cognitive impairment consistent with bilateral predominantly left frontal involvement and mild parietal involvement	
