# Supplementary material for: Uterus infantilis: a novel phenotype associated with AARS2 new genetic variants. A case report
Source: Front Neurol. 2023 Jun 29;14:878446. doi: 10.3389/fneur.2023.878446 (PMC10343430; doi:10.3389/fneur.2023.878446)
Supplement: Supplementary file 3 [file Data_Sheet_2.PDF]

Table 2. Activities of the respiratory chain enzymes, determined in a skeletal muscle biopsy sample		
Enzyme	Activity nmol/min/mg protein<br>citrate synthase	Range of normal activities nmol/min/mg protein<br>citrate synthase
NADH-dehydrogenase<br>(rotenone sensitive NADH-DH)	2.04<br>(0.321)	1.00 – 6.50<br>(0.375 – 1.00)
(NADH-cytochrome c reductase)<br>(rotenone sensitive)	0.928<br>(0.270)	0.100 - 0.490<br>(0.075 - 0.205)
(Succinate cytochrome c reductase)	0.030	0.020 - 0.100
(Cytochrome c oxidase)	0.112	0.150 -0.650
Citrate synthase	56	80–200 nmol/min/mg prot.
